# Dental amalgam exposure and multiple sclerosis: authors’ response

**DOI:** 10.1093/ije/dyag142

**Published:** 2026-08-19

**Authors:** Jie Guo, Tomas Olsson, Lars Alfredsson, Anna Karin Hedström

**Affiliations:** Department of Nutrition and Health, China Agricultural University, Beijing, China; Department of Clinical Neuroscience, Karolinska Institutet, Stockholm, Sweden; Department of Clinical Neuroscience, Karolinska Institutet, Stockholm, Sweden; Institute of Environmental Medicine, Karolinska Institutet, Stockholm, Sweden; Centre for Occupational and Environmental Medicine, Region Stockholm, Stockholm, Sweden; Department of Clinical Neuroscience, Karolinska Institutet, Stockholm, Sweden

We thank Tay and Blythe for their thoughtful comments regarding our study of dental amalgam exposure and multiple sclerosis (MS).^1^ We agree that the number of amalgam fillings may have reflected cumulative caries and restorative history, and that residual confounding by oral health cannot be excluded.

The estimates remained unchanged after adjustment for established MS risk factors and, in sensitivity analyses, for education, alcohol consumption, fish intake, and physical activity. Further adjustment for individual disposable income or household disposable income per family member had only a marginal influence on the estimates. Socioeconomic confounding should also be considered in the context of the Swedish dental-care system. Comprehensive dental care is provided free of charge to children and adolescents through a public dental-care system, with children routinely recalled for dental examinations.^2^ Thus, access to childhood dental care was less dependent on family income than in many other settings, although socioeconomic differences in caries risk and oral health may remain.

To address residual confounding by oral health, we conducted analyses by using the number of intact teeth, available for two-thirds of participants, as an indicator of oral-health history. The association between amalgam exposure and MS risk remained similar after this adjustment [odds ratio (OR) 1.35, 95% confidence interval (CI) 1.15–1.58 for amalgam fillings versus none]. Associations were observed within the strata of intact-tooth count, although some estimates were imprecise (Table 1). Intact-tooth count is an incomplete proxy for lifetime caries burden, but these findings suggest that differences in oral health alone are unlikely to explain the association. Moreover, tooth loss may partly lie downstream of long-term restorative history and dental treatment patterns, meaning that adjustment for intact teeth could also result in overadjustment.

**Table 1:** Associations between dental amalgam fillings and risk of MS, by number of intact teeth.

Amalgam	Total number of cases/controls	Number of intact teeth			
		<15	15–19	20–24	≥25
No	433/1027	1.0 (reference)	1.0 (reference)	1.0 (reference)	1.0 (reference)
Yes	1191/2222	1.54 (1.01–2.33)	1.72 (1.00–2.98)	1.45 (1.09–1.95)	1.29 (1.01–1.67)
OR for trend (95% CI)		1.12 (1.01–1.24)	1.15 (0.99–1.34)	1.20 (1.04–1.39)	1.12 (0.98–1.26)

Models were adjusted for age at disease onset, sex, residential area (according to study design), ancestry, smoking status at disease onset, past infectious mononucleosis, body mass index at age 20 years, and sun-exposure habits.

Tay and Blythe suggest that matching within 5-year age strata may have inadequately controlled for the marked birth-cohort gradient in amalgam use. We therefore repeated the analyses with adjustment for exact year of birth and the associations became stronger rather than weaker. Compared with participants without amalgam fillings, the adjusted ORs were 1.31 for 1–5 fillings, 1.50 for 6–10 fillings, 1.60 for 11–14 fillings, and 2.17 for ≥15 fillings, with an OR for trend of 1.19 (95% CI 1.12–1.26) (Table 2). Residual confounding by birth cohort therefore does not appear to explain the dose–response association.

**Table 2:** Associations between dental amalgam fillings and risk of MS, further adjusted for birth year.

Number of fillings	Cases/controls	**OR (95% CI)** ^a^	**OR (95% CI)** ^b^	OR for trend (95% CI)
0	742/1694	1.0 (reference)	1.0 (reference)	
1–5	560/1130	1.18 (1.02–1.35)	1.31 (1.13–1.52)	
6–10	597/1178	1.24 (1.07–1.43)	1.50 (1.26–1.77)	
11–14	283/539	1.31 (1.08–1.58)	1.60 (1.29–1.98)	
≥15	162/241	1.68 (1.33–2.12)	2.17 (1.67–2.83)	1.19 (1.12–1.26)

a Adjusted for age at disease onset, sex, residential area (according to study design), and ancestry.

b Adjusted for age at disease onset, sex, birth year, residential area, ancestry, smoking status at disease onset, past infectious mononucleosis, body mass index at age 20 years, and sun-exposure habits.

The proposed influence of an MS prodrome on dental healthcare use is also unlikely to account for the findings. Amalgam exposure represented cumulative lifetime restorative history rather than recent dental attendance. Among participants reporting many fillings shortly after symptom onset, it is implausible that more than a small proportion were placed during the proposed prodromal period. Estimates were similar when analyses were restricted to participants who completed the questionnaire within 1 year of symptom onset. In the progression analyses, the amalgam status was assessed at diagnosis and preceded disability outcomes by design.

Index-event bias is a theoretical concern in analyses restricted to individuals with MS. However, its magnitude depends on factors that influence both MS susceptibility and subsequent progression, and its direction and magnitude cannot be inferred from the case restriction alone. The progression models were adjusted for demographic and clinical characteristics, treatment exposure, smoking, body mass index, infectious mononucleosis, and sun-exposure habits. Further adjustment for HLA-DRB1*15:01 and HLA-A*02:01, which are the major established genetic determinants of MS susceptibility, did not attenuate the association (hazard ratio (HR) for confirmed disability worsening (CDW) 1.33, 95% CI 1.07–1.67 for at least six fillings compared with none). Although this analysis cannot exclude index-event bias arising from unmeasured factors, it provides no indication that selection through these major measured susceptibility factors explains the association.

Finally, Tay and Blythe compare the estimated between-group difference in the Expanded Disability Status Scale (EDSS) trajectory with thresholds used to define clinically meaningful within-person change. These quantities are not directly comparable. The β coefficient reflects differences in the mean longitudinal EDSS trajectories between the exposure groups, whereas conventional EDSS thresholds refer to changes within an individual (supplementary table S7 in the original manuscript). The longitudinal mixed-effects model was intended to complement the primary time-to-event analyses of confirmed disability worsening and progression to EDSS milestones.

We agree that our findings should not be interpreted as definitive evidence of causal effects. Rather, they support further investigation of mercury-related exposures in MS by using biomarker-based approaches and datasets with more detailed information on oral health and restorative materials.

## Ethics approval

The study was approved by the Regional Ethical Review Board at Karolinska Institute (reference numbers 2004–252/1–4 and 2013/1691–32) and was conducted in accordance with the ethical standards of the 1964 Declaration of Helsinki and its later amendments. All subjects in the study provided written informed consent before participation.

## Data Availability

Anonymized data underlying this article will be shared upon reasonable request from any qualified investigator who wants to analyse questions that are related to the published article.
